# Lactobacillus Kefiri LKF01 (Kefibios^®^) for Prevention of Diarrhoea in Cancer Patients Treated with Chemotherapy: A Prospective Study

**DOI:** 10.3390/nu13020385

**Published:** 2021-01-27

**Authors:** Michele Ghidini, Mariaceleste Nicoletti, Margherita Ratti, Gianluca Tomasello, Veronica Lonati, Mara Ghilardi, Maria Chiara Parati, Karen Borgonovo, Mary Cabiddu, Fausto Petrelli

**Affiliations:** 1 Oncology Unit, Fondazione IRCCS Ca’ Granda Ospedale Maggiore Policlinico, 20122 Milan, Italy; michele.ghidini@policlinico.mi.it (M.G.); gianluca.tomasello@policlinico.mi.it (G.T.); 2 Scientific Unit, Krea Innovazione srl, 20132 Milan, Italy; mariacelestenicoletti@gmail.com; 3 Oncology Unit, ASST of Cremona, 26100 Cremona, Italy; mratti.cremona@gmail.com; 4 Oncology Unit, ASST Bergamo Ovest, 24047 Treviglio, Italy; veronica_lonati@asst-bgovest.it (V.L.); mara_ghilardi@asst-bgovest.it (M.G.); mchiara.parati@gmail.com (M.C.P.); karen_borgonovo@asst-bgovest.it (K.B.); mary_cabiddu@asst-bgovest.it (M.C.)

**Keywords:** *Lactobacillus kefiri* LKF01, diarrhoea, chemotherapy, cancer

## Abstract

Diarrhoea is one of the main side effects that cancer patients face. The literature showsthat the incidence of chemotherapy (CT)-induced diarrhoea (grade 3–4) in treated patients is in the range of 10–20%, particularly after 5-fluorouracil (5-FU) bolus or some combination therapies of irinotecan and fluoropyrimidines. The aim of the present study was to evaluate the clinical effectiveness of *Lactobacillus kefiri* LKF01 (Kefibios^®^) in the prevention or treatment of CT-related diarrhoea in the cancer population. We conducted a prospective observational study. Patients enrolled were adults treated for at least four months with 5-FU-based CT. Kefibios^®^ was administered to patients every day. The primary outcome was the evaluation of the incidence of grade 3–4 CT-induced diarrhoea. We included 76 patients in the final analysis. A 6.6% incidence of high-grade diarrhoea was found in the evaluated population (4.7% of patients treated with 5-FU-based therapy and 8.5% of patients treated with capecitabine-based CT). The overall incidence of high-grade diarrhoea observed was higher in the 1st and 2nd cycles (3.9%), with a subsequent sharp reduction from the 3rd cycle (1.3%) and negativisation from the 5th cycle. *Lactobacillus kefiri* LKF01 (Kefibios^®^) is safe and effective in preventing severe diarrhoea in cancer patients receiving 5-FU or capecitabine-based treatment.

## 1. Introduction

Diarrhoea is a common complication of anticancer treatments, in particular chemotherapy (CT), radiotherapy (RT), and immunotherapy. CT-related diarrhoea is most commonly described with fluoropyrimidines (particularly 5-fluorouracil (5-FU) and capecitabine) and irinotecan [1,2]. Diarrhoea is dose limiting and represents the major toxicity of regimens containing a fluoropyrimidine with irinotecan (e.g., severe diarrhoea can rise to 15–20% with 5-fluorouracil, oxaliplatin and irinotecan (FOLFOXIRI) or 5- fluorouracil and irinotecan (FOLFIRI)-based CT [3,4,5]). Both 5-FU and irinotecan can cause acute damage to the intestinal mucosa, leading to a loss of epithelium. 5-FU induces the mitotic arrest of crypt cells, leading to an increase in the ratio of immature secretory crypt cells to mature villous enterocytes. The increased volume of fluid out of the small bowel exceeds the absorptive capacity of the colon, leading to clinically significant diarrhoea. CT-related diarrhoea can be debilitating and, in some cases, life threatening.

Patients may develop volume depletion, acute kidney injury, and electrolyte disorders. Infection, including sepsis, can happen due to damage of the intestinal mucosa, which is worsened in the setting of CT-induced neutropenia. Some patients need hospital admission for supportive measures. Treatment of diarrhoea consists of reducing bowel output with anti-diarrhoeal agents (e.g., loperamide), increasing liquid oral or parenteral intake, and normalising hydro-electrolytic disturbances. With this background, it is of paramount importance to ensure patient and caregiver education, as well as nurse and physician collaboration to implement pre-emptive measures and rapid treatment instauration.

Probiotics are live microorganisms that confer a health benefit when consumed in adequate amounts. When a microbial strain is indicated to be a probiotic, there are some specific prerequisites that need to be addressed. One of them is adhesion to the intestinal mucosa for colonisation and further interaction between the administered probiotic strains and the host. This specific interaction is required for the modulation of the antagonism against pathogens and for actions in the immune system. In healthy adults, probiotic administration increases the production of short-chain fatty acids, faecal moisture, frequency of defecation, and volume of stools [6].

The consumption of probiotics has increased over the years, and in particular, their use is approved for the prevention or treatment of acute, antibiotic-associated, and *Clostridium difficile*–associated diarrhoea; inflammatory bowel disease and irritable bowel syndrome; and reduction of risk for neonatal late-onset sepsis and necrotising enterocolitis [7]. Probiotics are a current strategy to treat dysbiosis, restoring microbial diversity and altering the perturbed intestinal microbiota with different and specific mechanisms of action. The main microorganisms used in the probiotics industry belong to the Lactobacillus and Bifidobacterium genera. A recent Cochrane review of 12 studies exploring the use of probiotics for prevention of treatment-related diarrhoea in cancer patients (CT or RT induced) showed limited evidence of benefit, mainly due to heterogeneity and underpowered studies [8]. However, probiotics may confer protective functions to the digestive system; they may prevent pathogen replication and toxin production due to competition for nutrients or sites of adhesion; and they also act on gut-associated lymphoid tissue modulation, inducing an increase in immunoglobulin A (IgA) production, activation of mononuclear cells, activation of lymphocytes, and production of cytokines [9]. Components of the probiotic metabolome (organic acids, bacteriocins, hydrogen peroxide, amines, etc.) have been reported to interact with multiple targets in some metabolic pathways that regulate cellular proliferation, differentiation, apoptosis, inflammation, angiogenesis, and metastasis. Some lactobacilli and bifidobacteria can produce antimicrobial peptides known as bacteriocins, which prevent the proliferation of selected pathogens. In particular, some of these compounds produced by *L. plantarum* and *L. acidophilus* have been shown to inhibit the growth of *Helicobacter*, *C. difficile*, rotaviruses, and multidrug-resistant *Shigella* spp. and *E. coli* in some gastrointestinal conditions. The enzymatic activities of probiotics in the gut lumen can play a role in the biological effects of these probiotics. Lactobacilli and bifidobacteria exhibit >20 different enzymatic activities, with β-galactosidase activity being the most typical.Secretory IgA (sIgA) is secreted by intestinal B cells and is expressed on the basolateral surface of the intestinal epithelium as an antibody transporter. sIgA facilitates the translocation of IgA dimers to the luminal surfaces of epithelial cells. Several studies have reported that probiotics show potent stimulation of the production of sIgA, thereby enhancing barrier function. Regardless, probiotics interact with intestinal and specific immune cells, resulting in the production of selected cytokines. Probiotics are capable of suppressing intestinal inflammation via the downregulation of TLR expression, secretion of metabolites that may inhibit tumour necrosis factor α (TNF-α) from entering blood mononuclear cells, and inhibition of nuclear factor kappa-light chain-enhancer of activated B cells (NF-κB) signaling in enterocytes. Through the mechanisms discussed above, probiotic bacteria restore the intestinal flora and have demonstrated a benefit in treating gastrointestinal diseases, including infectious diarrhoea in children, recurrent *C. difficile*-induced infection, and inflammatory bowel diseases [6]. Moreover, they may be beneficial in patients with CT-related diarrhoea [10].

Kefir grains are constituted by a complex symbiotic microbiota, and they are used to obtain fermented milks called “kefir”. One of the most important lactobacilli retrieved from kefir is *Lactobacillus kefiri* (LK). When LK was administered to healthy mice daily for 21 days, it increased IgA in faeces and reduced the expression of pro-inflammatory mediators in Peyer patches and mesenteric lymph nodes, where it also increased IL-10 [11]. When administered to healthy volunteers, LK modulated gut microbiota composition with a shift towards the reduction of several bacterial genera involved in the onset of pro-inflammatory response and gastrointestinal diseases [12].

We performed a prospective multicentre observational study aiming to evaluate the efficacy of LK in patients with cancer treated with 5-FU-based CT (RT). We postulated that by providing LK in the form of Kefibios^®^ from the start and during CT (RT), we could reduce by 10% the absolute risk of severe diarrhoea (CTCAE grade [G] 3–4) to an absolute incidence of 5–10%.

## 2. Materials and Methods

### 2.1. Participants and Study Design

This was a prospective observational study conducted at two oncology centres in Lombardy, Italy (ASST Bergamo Ovest and ASST Cremona). Participants were adult patients who had undergone any CT doublet containing a fluoropyrimidine analogue for curative (adjuvant) or palliative (metastatic) treatment of at least four months duration. Treatment could have included concomitant abdominal RT.

The intention was to treat 80 patients; 78 patients were planned from February 2018 to September 2019; at the end, 76 patients were analysed. Median age was 67 years. There were 50 males and 28 females. Patients with any solid tumour who started fluoropyrimidine-based CT and without baseline diarrhoea were recruited. Informed consent was obtained from each participant upon recruitment. Patients consuming probiotics at the moment of CT initiation were not considered. Toxicity was evaluated every cycle for a maximum of 4–6 cycles (according to the schedule received) in a treatment period of four months. Observation was continued up to six months to check for further adverse events (AEs) of the study drug or severe adverse events (SAEs).

### 2.2. Treatment

*Lactobacillus kefiri* (Kefibios^®^) LKF01 (DSM 32079) powder was contained in capsules, and then, the content was mixed in a vegetable oil solution. On first use, it was necessary to disperse the powder contained in the special capsule inside the vial, paying particular attention to keep the capsule upright during the opening manoeuvre. Then, the solution had to be shaken well before use and before any other subsequent use. Kefibios^®^ was consumed every day (5 drops per day) with an empty or full stomach, before or after a meal with some liquid. Five drops of Kefibios^®^ include at least 1 billion vital microorganisms.

### 2.3. Study Objectives and Endpoints

The primary study aim was to evaluate the incidence of severe acute diarrhoea (grade 3–4) during fluoropyrimidine-containing poly-CT (associated or not with abdominal RT). Secondary objectives included the following:

1. The overall incidence of diarrhoea.
2. Drugs used for its treatment.
3. The risk of infections/sepsis and severe neutropenia or febrile neutropenia.
4. Any dose reductions/interruptions of the current treatment.
5. The effects on anti-EGFR-induced acneiform rash (where applicable).
6. Any AEs of the treatment.

The primary endpoint was evaluation of the incidence of common terminology criteria for adverse events (CTCAE) grade [G] 3–4 CT-induced diarrhoea. Secondary endpoints included rate of G1–4 diarrhoea, rate of dose reduction/interruption, use of symptomatic therapies, rate of infections/sepsis or severe or complicated neutropenia, rate of severe anti-epidermal growth factor receptor (EGFR)-related skin rash, AEs of treatment, and treatment adherence.

### 2.4. Statistical Analysis

The intention-to-treat (ITT) population included all patients who received at least one dose of Kefibios^®^ and was used to evaluate the safety profile of Kefibios^®^ in this study. The per-protocol (PP) population included all patients who took at least one dose of Kefibios^®^ and had at least one post-baseline effectiveness measurement. The incidence of severe diarrhoea (G3–4) in advanced cancer patients treated with different fluoropyrimidine-based CT doublets or triplets is approximately 10–20% depending on type of agents (FOLFOX/FOLFIRI vs. FOLFIRI/FOLFOX + anti-EGFR or FOLFOXIRI + bevacizumab). A dropout rate of six months for progression, death, switching to another therapeutic modality, or toxicity has been hypothesised for about 10–30% of patients depending on the therapeutic schemes. In this way, a population of approximately 100 patients could be sufficient to demonstrate a difference of approximately 10% (50% reduction) in the rate of severe diarrhoea (e.g., approximately 15–20% to 5–10%) with a power of 0.90 at a significance level of 0.05 in a two-tailed (chi-square) zeta test. Counts and percentages were used for categorical variables. Continuous variables were presented as descriptive statistics. AEs (e.g., diarrhoea) were reported as number and rates and summarised by severity (G1–4 vs. G3–4) time during treatment. The main results were evaluated up to the 6th cycle.

In all statistical analyses, *p* < 0.05 (two-sided test) was considered significant.

## 3. Results

### 3.1. Flow of Patients

A total of 78 patients were enrolled from February 2018 to September 2019. All patients except one who died after one cycle of CT were evaluable for the PP and ITT analysis. All patients were in good clinical condition (Eastern Cooperative Oncology Group [ECOG] performance status 0–1). Among patients, 54 had colorectal or anal cancers, 16 had gastro-oesophageal carcinomas, six had pancreatic adenocarcinoma, one had cancer with an unknown primary, and one had breast cancer. Patients had localised/locally advanced disease and received (neo) adjuvant therapies (n = 43). In 35 cases, patients had metastatic cancer. Treatment received consisted of oxaliplatin-based combinations (FLOT, FOLFOX, or XELOX with or without targeted therapies) in 55 patients, irinotecan-based CT (e.g., FOLFIRI plus or minus anti-EGFR or anti-VEGF) in six patients, CT triplets (FOLFIRINOX or FOLFOXIRI alone or in combination) in 13 patients, and other combinations in four patients. Concentration of 5-FU was 400 mg/m^2^ bolus every 15 days plus continuous infusion for 46 h of 2400 mg/m^2^ every 15 days for doublets and 3200 mg/m^2^ of 5-FU in continuous infusion for 46 h every 14 days for triplets. All patients received Kefibios^®^ every day. Most patients received one, two, three, four, five, and six CT cycles. Two patients were not evaluable due to diarrhoea present before start of therapy (short gut syndrome) and due to death after the 1st cycle. Three hundred and seventy-six cycles were evaluated.

### 3.2. Severe Diarrhoea

Globally, the incidence per cycle of G3–4 diarrhoea was 3.9% at the 1st cycle, 3.9% at the 2nd cycle, 1.3% at the 3rd cycle, 1.5% at the 4th cycle, and 0% at the 5th and 6th cycle (mean 1.7%) (Table 1). Amongst 76 patients evaluable for efficacy, five (6.5%) had G3–4 diarrhoea. Seventeen patients did not develop diarrhoea at all up to six cycles (22%).

### 3.3. Secondary Endpoints

**Figure 1:**
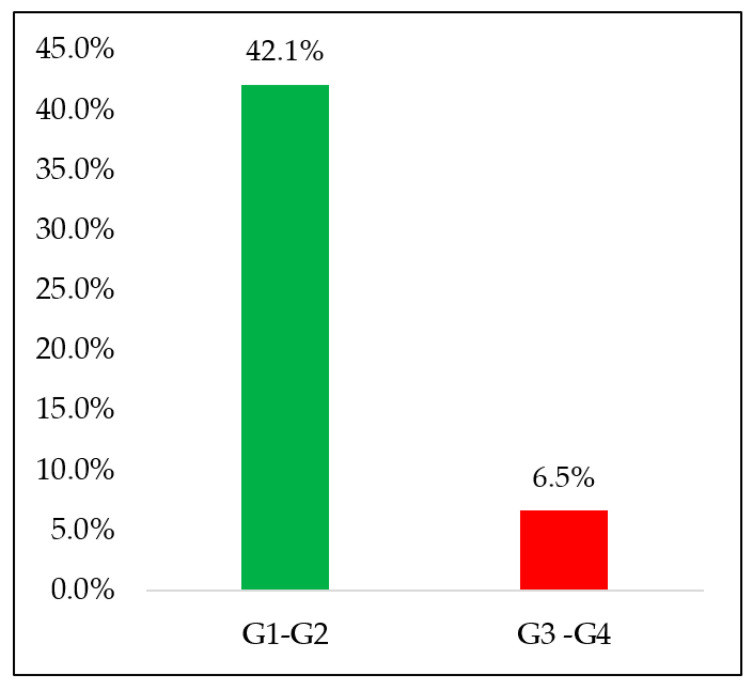
Percentage of patients with severe (G3–4) or mild (G1–2) diarrhoea.

Overall, the rate of G1–4 diarrhoea was 35.5% at cycle 1, 34.0% at cycle 2, 28% at cycle 3, 27.6% at cycle 4, 20.0% at cycle 5, and 25.0% at cycle 6. Mean (per cycle) incidence of diarrhoea was 28.3% along the entire treatment period (six cycles) (Table 2). Globally, 48.7% of patients (n = 37) had some form of diarrhoea (n = 109 episodes of diarrhoea, of which 72% occurred within 3 cycles), as shown in Figure 1.

CT was interrupted before six cycles in seven patients (n = 2 due to personal choice, n = 2 due to death or progression of disease, n = 2 due to medical decision, and n = 1 due to G3 diarrhoea (1.3%)). Some dose reduction (for 5-FU/capecitabine, all drugs, or with omission of oxaliplatin) was performed in 38 patients, but in 86% of cases (n = 33), it was for reasons other than diarrhoea (neuropathy, haematological or cardiovascular toxicities, infection). Only five patients received anti-EGFR agents, with two episodes of mild–moderate skin toxicity (40%). Severe neutropenia or febrile neutropenia was observed in 5.2% of patients, with one patient who was hospitalised for pneumonia.

No severe cutaneous rashes were observed. Compliance was good (all patients assumed treatment for four months). No side effects were related to Kefibios^®^.

### 3.4. Subgroup Analysis

According to the type of agent used for treatment (cisplatin/oxaliplatin vs. irinotecan-based CT doublets/triplets), the rate of G3–4 diarrhoea was 1.7%, 1.7%, 1.8%, 0%, 0%, 0%, and 0% versus 11.0%, 11.0%, 0%, 0%, and 0% at the 1st, 2nd, 3rd, 4th, 5th, and 6th cycle (mean 0.86 vs. 3.6%). Among all patients that suffered some grade of diarrhoea, 27 had toxicity at the 1st cycle (73%) and 10 had toxicity at the 2nd cycle (27%). All grades of diarrhoea were 35% (3% of G3–4 diarrhoea) with doublets (plus or minus biologics) and 69% (15.3% G3–4) with CT triplets (plus or minus biologics). Splitting 5-FU and capecitabine-based CT, rates of G3–4 diarrhoea were 4.7%, 4.7%, 0%, 0%, 0%, and 0% vs. 2.9%, 2.9%, 2.9%, 0%, 0%, and 0% at cycle 1, 2, 3, 4, 5, and 6, respectively (Table 3).

**Figure 2:**
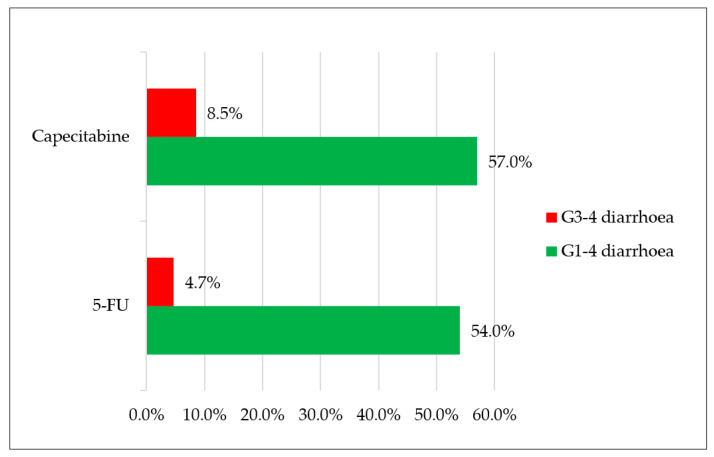
Percentage of patients with G1–4 diarrhoea and G3–4 diarrhoea receiving chemotherapy treatment with capecitabine or 5-fluorouracil (5-FU).

**Figure 3:**
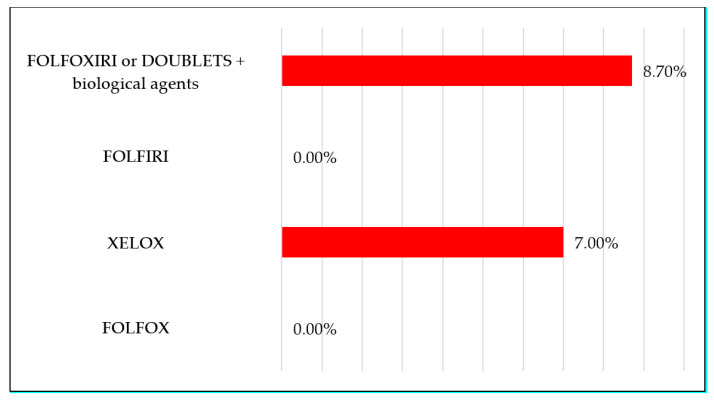
Percentage of observed G3–4 diarrhoea for various treatments.

Overall, 54% and 4.7% of evaluable patients treated with 5-FU-based CT had G1–4 and G3–4 diarrhoea, respectively, during treatment; conversely, 57% and 8.5% of patients treated with capecitabine-based CT had the same toxicities (Figure 2). Furthermore, based on the four classes of therapeutic regimens more commonly used in clinical practice, the observed rates of G3–4 diarrhoea were 0% for FOLFOX and FOLFIRI, 7% for XELOX, and 8.7% for FOLFOXIRI or doublets plus biological agents (Figure 3). Patients with colorectal cancer had a 48% rate of diarrhoea (all grades) and 2% of G3–4 events. Differently, gastro-oesophageal cancer patients registered a 56% rate of diarrhoea with a higher prevalence of severe events (12.5% G3–4).

Table 4 summarises the main findings of our study.

## 4. Discussion

The role of probiotics in the prevention of CT-related diarrhoea has been a matter of debate for many years. Evidence is poor due to the limited number of multicentre randomised controlled trials (RCTs) and heterogeneity of the available series. The recent Cochrane systematic review included RCTs investigating the effect of probiotics for prevention or treatment of CT- or RT-related diarrhoea. The studies were underpowered and heterogeneous; moreover, the effect of probiotics on the prevention of CT-related diarrhoea could have been underestimated because of concomitant administration of RT in many of the included studies, with potential additive gastrointestinal toxicity [7]. Differently, a meta-analysis of 13 RCT studies evaluating the role of probiotics in preventing CT-related diarrhoea found a statistically significant reduction in the total diarrhoea rate (risk ratio (RR) = 0.47, 95% confidence interval (CI) [0.35, 0.63], *p* < 0.00001) and G3–4 diarrhoea (RR = 0.16, 95% CI [0.05, 0.42], *p* = 0.0008). Moreover, the duration of AEs when probiotics were added to conventional symptomatic treatment was significantly shortened (median duration (MD) = −1.92, 95% CI [−11.96, −11.88], *p* < 0.00001). However, in patients with G1–2 diarrhoea, the difference was not statistically significant (RR = 0.81, 95% CI [0.53, 1.24], *p* = 0.34) [13].

*Lactobacillus kefiri* intake has been associated with a significant modification in the gut microbiota of healthy volunteers. Indeed, even after a single month of oral intake, a reduction in the faecal bacterial load of several bacterial genera was observed. In particular, bacterial strains directly associated with pro-inflammatory responses and gastrointestinal pathologies such as chronic inflammatory bowel diseases were significantly reduced in faecal load at the end of probiotic administration if compared to baseline samples [12]. *Lactobacillus kefiri* exerted an anti-inflammatory effect by inducing the mucosal immune response, with documented increase of IgA in healthy Swiss mice and downregulation of pro-inflammatory cytokines in Peyer patches and mesenteric lymph nodes [11]. Surely, lactobacillus’ anti-inflammatory properties may play a role in the control of CT-induced toxicities such as diarrhoea, which is a consequence of an iatrogenic colitis. Some initial data also report a selective pro-apoptotic effect of *Lactobacillus kefiri* on human gastric cancer cells. The effect was shown to be dose-dependent, with a peak of 66.3% of apoptotic rate at a concentration of 5.0 mg/mL [14].

In our series, we recorded 6.6% of high-grade diarrhoea, with 4.7% for 5-FU-based treatments and 8.5% for capecitabine-based CT; 22% of patients did not complain about this AE during treatment. These percentages were lower than the values reported in a meta-analysis evaluating the incidence and relative risk of G3–4 diarrhoea in patients treated with capecitabine (17%) or 5-FU (12.9%) [5]. Irinotecan-based treatments had a higher incidence of high-grade diarrhoea, with a mean rate of 3.6% compared with 0.86% recorded for oxaliplatin-based treatments. This report is consistent with the findings of the previously cited meta-analysis, where the RR for high-grade diarrhoea was doubled for patients receiving irinotecan compared with oxaliplatin (2.35 vs. 1.40). In particular, with the use of *Lactobacillus kefiri*, no events of high-grade diarrhoea were registered during treatment with FOLFOX or FOLFIRI. We observed a 7% rate of G3–4 diarrhoea with XELOX and 8.7% with FOLFOXIRI or doublets plus biological agents. Therefore, prevalence of high-grade diarrhoea was considerably lower than reported in the literature, with a 10% rate for FOLFOX and FOLFIRI, 17% for XELOX, and 18–20% for FOLFOXIRI or doublets in association with biological agents [3,4,5]. Interestingly, in our series, the overall incidence of high-grade diarrhoea was higher at the 1st and 2nd cycles (3.9%), with a subsequent sharp reduction from the 3rd cycle (1.3%) and negativisation from the 5th cycle. A possible explanation may be found in the high rate of dose reduction applied in the cohort of evaluated patients (50%). Although dose reduction was caused by toxicities different from diarrhoea in most of the cases (86% for neuropathy, haematological or cardiovascular toxicities, infection), dose adjustments might have positively influenced the incidence of gastrointestinal toxicities. In addition, proper symptomatic treatment for diarrhoea (i.e., loperamide assumption) could have been delayed and started later than the first occurrence of the AE. As a result of the early onset of diarrhoea during treatment, anticipating the oral administration of *Lactobacillus kefiri* may be hypothesised, with the oral intake starting before the first cycle of CT and not concomitantly as we did in our study. *Lactobacillus kefiri* was recovered in the faeces of healthy volunteers after one month of probiotic intake. Moreover, after one month, the gut microbiota composition was significantly modified, with reduction of several bacteria genera associated with pro-inflammatory response and gastrointestinal diseases [12]. Therefore, in cancer patients who are candidates for CT, one month of probiotic oral intake prior to the beginning of treatment could possibly reduce the high rates of early-onset diarrhoea. Recent guidelines on the management of cancer treatment-related mucositis suggested the use of probiotics containing Lactobacillus for the prevention of diarrhoea during CT-RT and RT for pelvic cancers. Recommendations were based on two RCTs [15]. The first study evaluated the use of Bifilact^®^ probiotics (Lactobacillus acidophilus LAC-361 and Bifidobacterium longum BB-536) in patients with pelvic malignancies treated with RT (46%) and CT-RT (54%). Bifilact^®^ was effective in reducing the incidence of high-grade diarrhoea only in patients who received pelvic surgery and standard doses of probiotics (but not high doses) [16]. A second study aimed to determine the effectiveness of the probiotics in the prevention of irinotecan-induced diarrhoea due to reduction of intestinal beta-d-glucuronidase activity in patients with colorectal cancer. Patients were randomised to receive either the probiotic formula Colon Dophilus^™^ or placebo. The administration of probiotics compared with placebo led to a reduction in the incidence of severe diarrhoea of grade 3 or 4 (0% for probiotic vs. 17.4% for placebo, *p* = 0.11), as well as reduction of the overall incidence of diarrhoea (39.1% for probiotic vs. 60.9% for placebo, *p* = 0.24). Moreover, the incidence of enterocolitis was reduced (0% for probiotic vs. 8.7% for placebo) [17]. Many factors limit the evidence and prevent making strong recommendations regarding the use of probiotics. First, studies are heterogeneous, they use different bacterial strains and doses of bacteria, and incidence of diarrhoea varies depending on the use of surgery, CT, RT, or a combination.

We acknowledge some limitations linked to our study. We chose a prospective single-arm design without randomisation or a control arm, two centres recruited patients, and the number of included cases is limited. Moreover, our case series was heterogeneous for type of treatment and histologies of the included tumours. However, differently from most of the previously conducted trials, we decided to include patients treated with CT only.

## 5. Conclusions

Our study provides evidence that the use of *Lactobacillus kefiri* LKF01 is safe and reduces the incidence of diarrhoea compared with historical data reported in the literature for cancer patients receiving a 5-FU or capecitabine-based treatment. However, a larger RCT is warranted to strengthen the role of probiotics in the prevention of CT-induced diarrhoea. Moreover, the hypothesis of a preventive oral intake of *Lactobacillus kefiri* for one month prior to CT initiation is intriguing but requires further evaluation.

## Figures and Tables

**Table 1:** Incidence of grade 3–4 diarrhoea out of the total population evaluated.

Cycle	Incidence of G3–4 Diarrhoea
1	3.9%
2	3.9%
3	1.3%
4	1.5%
5	0.0%
6	0.0%
Mean	1.7%

**Table 2:** Total incidence of grade 1–4 diarrhoea out of the total population evaluated.

Cycle	Incidence of G1–4 Diarrhoea
1	35.5%
2	34.0%
3	28.0%
4	27.6%
5	20.0%
6	25.0%
Mean	28.35%

**Table 3:** Rates of grade 3–4 diarrhoea for different therapeutic agents and combinations.

Cycle	Cisplatin/Oxaliplatin	Doublets and Triplets of Irinotecan	5-FU	Capecitabine
1	1.7%	11.0%	4.7%	2.9%
2	1.7%	11.0%	4.7%	2.9%
3	1.8%	0%	0%	2.9%
4	0%	0%	0%	0%
5	0%	0%	0%	0%
6	0%	0%	0%	0%
Mean	0.86%	3.6%	4.7%	1.45%

**Table 4:** Main findings of the study.

Characteristics	Main Findings
Type of study	Prospective observational
Number of patients	78 (76 evaluable)
Median age	67 years
Treatment duration	4 months
Probiotic used	*Lactobacillus kefiri*
Disease (number):	
GASTRO-OESOPHAGEAL	16
COLORECTAL	51
PANCREATIC	5
OTHER	6
Stage of disease (%):	
localised	55
metastatic	45
Overall diarrhoea G1–4, mean per patient (%)	48.7
Overall diarrhoea G3–4, mean per patient (%)	6.5
Diarrhoea G3–4 according to schedule used, mean per patient (%):	
FOLFOX	0
XELOX	7
FOLFIRI	0
FOLFOXIRI (or doublets) ± biological agents	8.7
CAPECITABINE-BASED	8.5
5FU-BASED	4.7
Compliance to treatment (dose assumed, %)	100
Adverse Events (number)	0

## Data Availability

The data presented in this study are available on request from the corresponding author. The data are not publicly available due to privacy policy.
